# mMCP7, a Mouse Ortholog of δ Tryptase, Mediates Pelvic Tactile Allodynia in a Model of Chronic Pelvic Pain

**DOI:** 10.3389/fpain.2021.805136

**Published:** 2022-01-12

**Authors:** Goutham Pattabiraman, Zhiqiang Liu, Madhumita Paul, Anthony J. Schaeffer, Praveen Thumbikat

**Affiliations:** Department of Urology, Feinberg School of Medicine, Northwestern University, Chicago, IL, United States

**Keywords:** mast cells (MC), pelvic pain in men, prostatitis, neuroimmune activation, tryptase (TPS)

## Abstract

Chronic prostatitis/Chronic pelvic pain syndrome (CP/CPPS) is a condition that affects a large number of men and has unknown etiology. We have previously demonstrated the presence of elevated levels of mast cell tryptase in expressed prostatic secretions (EPS) of CP/CPPS patients. In a murine model of CP/CPPS, we showed tryptase and its cognate receptor PAR2 as critical to the development of pelvic pain and lower urinary tract symptoms. Here, we extend these observations to demonstrate that an isoform of tryptase called delta (δ)-tryptase, is elevated in the EPS of patients with CP/CPPS and is correlated with pelvic pain symptoms. Using an *Escherichia coli* (CP1) -induced murine model of CP/CPPS, we demonstrated a differential response in C57BL/6J and NOD/ShiLtJ mice, with C57BL6/J mice being resistant to an increase in pelvic tactile allodynia, despite having equivalent levels of activated mast cells in the prostate. Activated tryptase^+ve^ mast cells were observed to be in closer apposition to PGP9.5^+ve^ nerve fibers in the prostate stroma of NOD/ShiLtJ in comparison to C57BL/6J mice. The mouse ortholog of δ-tryptase, mouse mast cell protease 7 (mMCP7) has been reported to be unexpressed in C57BL/6J mice. We confirmed the absence of mMCP7 in the prostates of C57BL/6J and its presence in NOD/ShiLtJ mice. To evaluate a role for mMCP7 in the differential allodynia responses, we performed direct intra-urethral instillations of mMCP7 and the beta (β)-tryptase isoform ortholog, mMCP6 in the CP1-infection model. mMCP7, but not mMCP6 was able to induce an acute pelvic allodynia response in C57BL/6J mice. *In-vitro* studies with mMCP7 on cultured mast cells as well as dissociated primary neurons demonstrated the ability to induce differential activation of pain and inflammation associated molecules compared to mMCP6. We conclude that mMCP7, and possibility its human ortholog δ-tryptase, may play an important role in mediating the development of pelvic tactile allodynia in the mouse model of pelvic pain and in patients with CP/CPPS.

## Introduction

Prostatitis is a frequent urologic diagnosis in men under the age of 50, accounting for 8% of all office visits to urologists and an estimated 1% of all primary care visits in the US (1, 2). The majority (over 90%) of prostatitis cases are classified as chronic prostatitis / chronic pelvic pain syndrome (CP/CPPS, NIH Category III). CP/CPPS is a disorder characterized by severe pain originating from the pelvic region and may be associated with lower urinary tract symptoms (3). While the immune and nervous systems are believed to play a critical role in the development of this chronic pain that is characteristic of CP/CPPS, the interplay and the crosstalk between the two systems remain elusive.

Mast cells are a multifaceted and multifunctional immune cell that upon activation releases a plethora of pro-inflammatory mediators such as cytokines and chemokines as well as pain mediators including histamine, proteases (e.g., tryptases), serotonins, neurotrophins, neurotransmitters, and prostaglandin E2 (4–10). Mast cells also contain preformed pockets of immune mediators and neurotrophins, such as histamine and nerve growth factor (NGF) that can be rapidly released upon activation (11, 12). The rapid release of these factors from mast cells provides a possible mechanism for the peripheral sensitization and increased neuronal excitability seen in CP/CPPS. We have previously shown that mast cells play an important role in the development of pain using a non-infectious autoimmune driven murine model—experimental autoimmune prostatitis (EAP) (13, 14). Proteases released from mast cells act on protease-activated receptor 2 (PAR2) on sensory neurons and nerve fibers (among other cell types) to play an integral role in the development of nociception (15–17).

Expressed prostatic secretions (EPS) from patients with CP/CPPS show increased levels of β-tryptase among other inflammatory markers like NGF, MCP-1, MIP-1α, IL-17, and IL-7 (13, 14, 18–20). Humans express five different isoforms of the tryptase gene clustered on chromosome 16p13.3. TPSG1 encodes gamma (γ)-tryptase, TPSB2 encodes beta (β)II and βIII-tryptase, TPSAB1 codes for alpha (α)-tryptase and βI-tryptase, TPSD1 accounts for delta (δ)-tryptase, and TPSE1 encodes epsilon (ε)-tryptase (21, 22). β-tryptase is the most abundant and most studied of these tryptases. In the humans, δ-tryptase while maintaining an 80% similarity to α- and β-tryptases, is a truncated isoform and is thought to be a catalytically inactive form (23, 24). However, one report suggests that a recombinant δ-tryptase protein maintains its proteolytic activity to cleave a trypsin-sensitive substrate D-Ile-Phe-Lys pNA, albeit to a lesser extent than β-tryptase (22).

We have previously reported the ability of an uropathogenic clinical prostate isolate of *Escherichia coli* (CP1) to colonize the prostates of mice and establish pelvic tactile allodynia in male NOD/ShiLtJ mice. In the mouse model of CP/CPPS upon CP1 instillation, we observe increased tactile allodynia in NOD/ShiLtJ mice, but not in C57BL/6 mice. Interestingly, while NOD/ShiLtJ and C57BL/6 mice express most of the same proteases including mouse mast cell protease-6 (mMCP6), a mouse ortholog of the human β-tryptase, C57BL/6 mice have a deficiency in the mouse ortholog of δ-tryptase mast cell protease 7 (mMCP7) gene with a point mutation in the exon/intron 2 splice of the mMCP7 gene rendering them unable to express mMCP7 protein (25). While both these tryptases are quite homologous, the expression of the two tryptases is differentially regulated (26, 27). Furthermore, mMCP-6 and mMCP-7 are functionally distinct tryptases by multiple criteria and trigger differential effects (28).

In this study, we observe the increased levels of δ-tryptase in the EPS of CP/CPPS patients. In addition to verifying the absence of mMCP7 in C57BL/6 mice, we determined the effect of administration of mMCP7 on pelvic tactile allodynia in C57BL/6 mice. Furthermore, we explore the role played by mMCP7 in mediating activated mast cell interaction with neural structures in the prostate and mediating pelvic tactile allodynia in the *E. coli* CP1-induced mouse model of CP/CPPS. This study demonstrates the role played by mMCP7 in driving pelvic tactile allodynia responses and suggests a new and unappreciated role for δ-tryptase in chronic pelvic pain.

## Materials and Methods

### Ethics Statement

Animal studies were conducted under protocols ISO00003413 and ISO00003638 approved by the Institutional Animal Care and Use Committee (IACUC) at Northwestern University. IACUC at Northwestern is AAALAC accredited. Northwestern University has an Animal Welfare Assurance on file with the Office of Laboratory Animal Welfare (A3283-01). Northwestern University conducts its reviews in accordance with United States Public Health Service (USPHS) regulations and applicable federal and local laws. The composition of the IACUC meets the requirements of the USPHS policy and the Animal Welfare Act Regulations.

### Patient Sample Collection

Appropriate National Institute for Health—Chronic Prostatitis Symptom Index (NIH-CPSI) questionnaire data, expressed prostatic secretions (EPS) samples were collected by Dr. A. Schaeffer and colleagues at the department of urology outpatient clinic at Northwestern Memorial Hospital, Chicago IL (NCT01676857). The written consent procedure was reviewed by the Northwestern University Institutional review board Panel D and approved with the IRB number STU00030121. The Northwestern University Institutional review board Panel D specifically approved this study with IRB number STU00030121. All participants provided written informed consent to participate in this study. The questionnaire has been designed specifically to assess patient scored symptoms of CP/CPPS and divides score into three areas: Pain, Urinary and Quality of life. EPS samples were immediately stored at −80°C for future analyses.

### Mice

Male C57BL/6, and NOD/ShiLtJ mice (5–7 weeks of age) were purchased from Jackson Laboratory (Bar Harbor, ME, USA). Mice were housed in a single environmentally controlled room within the Northwestern University animal facility. All animal experiments and procedures have been approved by the Northwestern University Animal Care and Use Committee. All pain-related animal experiments were conducted in accordance with IASP Guidelines for the use of animals in research issued on January, 2014.

### *In vitro* Recombinant Mast Cell Proteases Maturation

Recombinant mouse mast cell protease 6 (mMCP6) and mouse mast cell protease 7 (mMCP7), also called mouse tryptase beta-1, were purchased from R&D systems (Minneapolis, MN, USA). mMCP6 was matured following the manufacturer's instructions using bacterial thermolysin protein (R&D systems, Minneapolis, MN, USA). Briefly, mMCP6 and bacterial thermolysin were diluted to 200 and 0.2 μg/mL respectively in maturation buffer (50 mM Tris, 10 mM CaCl_2_, 150 mM NaCl, 0.05% (w/v) Brij-35, pH 7.5). 25 μL of 200 μg/mL mMCP6 was combined with 25 μL of 0.2 μg/mL bacterial thermolysin and incubated at room temperature for 15 mins for maturation of mMCP6. Maturation was stopped using 50 μL of 10 mM 1,10-phenanthroline (Sigma-Aldrich, Inc., St. Louis, MO, USA). mMCP6 and mMCP7 are diluted in PBS or media to desired concentration.

### Intra-Urethral Instillations

Mouse intra-urethral instillations with CP1 and recombinant mast cell proteases were performed as described previously (29–31). Briefly, CP1 *E. coli* bacteria were grown in LB overnight shaking at 37°C, followed by overnight static subculture at 37°C. The next day, bacteria were concentrated at 2 × 10^10^ bacterial/mL in PBS, and 10 μL (2 × 10^8^ bacteria) was instilled intra-urethrally into isoflurane-anesthetized male C57BL/6 and NOD/ShiLtJ mice. Age-matched control male mice received an intra-urethral instillation of PBS (Gibco, Paisley, UK) and were kept in separate cages.

Intra-urethral instillation of mouse mast cell proteases was also performed as described above. Mature recombinant mMCP6 prepared above and recombinant mMCP7 were diluted in PBS to a final concentration of 1 ng/mL, and 10 μL was instilled intra-urethrally into isoflurane-anesthetized male C57BL/6 mice. Age-matched control male C57BL/6 mice received an intra-urethral instillation of PBS and were kept in separate cages.

### Bone Marrow Mast Cell Isolation, Differentiation and Stimulation

Bone marrow mast cell (BMMC) isolation and differentiation was performed as previously described (32). Briefly, the femur and tibia of C57BL/6 mice were collected and the muscles were removed, and then the bone marrow (including the stem cells and progenitor cells) were flushed from the tibia and femur with repeated injections of ice cold PBS with a syringe using aseptic techniques. The harvested cells were then washed twice with PBS, and then resuspend and differentiated in culture media [RPMI 1,640 medium containing 2 mM L-glutamine, 15% FCS, 1 x Penicillin/streptomycin, 10 mM HEPES (all from Thermo Fisher, Hampton, NH, USA), 0.07% β-mercaptoethanol (Sigma, St. Louis, MO, USA), and 30 ng/mL recombinant IL-3 (BioLegend^®^, San Diego, CA, USA)]. Fresh media was added every third day to the culture and the differentiation of BMMC was completed after 6 weeks of culture. Differentiation was monitored by FACS analysis (described under flow cytometer analysis).

After 6 weeks of differentiation, the isolated BMMCs were stimulated with mature recombinant mMCP6 prepared above and recombinant mMCP7 for specified times. BMMCs were lysed for RNA extraction and real-time quantitative reverse-transcriptase PCR.

### Real-Time Quantitative Reverse-Transcriptase PCR

Total RNA was isolated using RNeasy Mini Kit (Qiagen, Valencia, CA, USA), and cDNA synthesis, starting with 1 μg of total RNA, was performed with random hexamers using High-Capacity cDNA Reverse Transcription Kit (Life Technologies Corporation, Grand Island, NY, USA) per the manufacturer's instructions. Primers for quantitative PCR (qPCR) were designed for the genes of interest using the NIH online primer blast tool. qPCR reactions were performed using SsoAdvanced™ universal SYBR^®^ green (Bio-Rad, Hercules, CA, USA) and run on the CFX Connect (Bio-Rad, Hercules, CA, USA) platform. The following primers were used —mMCP7: 5′-CGCACTACTCCTCACTGTGT-3′ (forward), 5′-GTGTCATAGCTGGACCGGG-3′ (reverse); mMCP6: 5′-GGAGGTTCTCTCATCCATCCAC-3′ (forward),5′-CCTGTTCAAAGAGAGGAGCTGG3′ (reverse);NGFβ:5′- GTTTTGCCAAGGACGCAG CTTTC-3′ (forward),5′-GTTCTGCCTGTACGCCGATCAA-3′ (reverse);KNG1:5′-GCCAACTTCTCA CAGAGCTGTAC-3′ (forward), 5′- TGACCAAGCACCTCCTTCAGCT-3′ (reverse); and L19: 5′-CAACTCCCGCCAGCAGAT-3′ (forward), 5′-CCGGGAATGGACAGTCACA-3′ (reverse). The data were analyzed by the 2−ΔΔCT method (33), and normalized to L19 as the housekeeping gene.

### Western Blotting

Prostate samples were lysed in 1X RIPA lysis buffer (Santa Cruz Biotechnology, Dallas, TX, USA) containing complete-EDTA protease inhibitor cocktail and phosSTOP phosphatase inhibitor (Millipore-Sigma, Burlington, MA, USA). Cell lysates were cleared by centrifugation at 14,000 rpm for 30 min at 4°C, and insoluble debris was discarded. Proteins were separated by SDS-PAGE on Criterion 4–20% mini gels (Bio-Rad, Hercules, CA, USA), transferred to polyvinylidene fluoride membranes (Bio-Rad, Hercules, CA, USA), blocked, and probed with the respective Abs. Immunoblotting was performed using the following antibodies—rabbit anti-tryptase alpha/beta 1 (catalog # 142648, United States Biological, Salem, MA, USA) and developed using SuperSignal^TM^ west -pico or -femto chemiluminescence kit (Thermo Fisher, Hampton, NH, USA).

### Flow Cytometry

Undifferentiated bone marrow cells and 6-week differentiated BMMCs were washed twice with FACS buffer (2% FCS in PBS). Cells were pre-incubated with TruStain FcX™ (anti-mouse CD16/32) antibody (catalog # 101319, RRID:AB_1574973; BioLegend®, San Diego, CA) before being stained with APC anti-mouse FcεRIα (catalog # 134315, RRID:AB_10640726; BioLegend®, San Diego, CA), and PE anti-mouse CD117 (c-Kit) (catalog # 12-1171-82, RRID:AB_465813; Thermo Fisher, Hampton, NH, USA). Samples were run on a BD Accuri C6 ™ (BD Biosciences, San Jose, CA, USA) cytometer and analyzed using FlowJo™. Cells were then gated for forward and side scatter followed by gating for single cells. Mast cells were identified by double positive staining of CD117 and FcεRIα (**Figure 4A**).

### Immunofluorescence and Imaging

At the endpoint of CP1 instillation experiment, the mice were euthanized, and the prostates were harvested from mice as described previously (34). Prostate samples were fixed in 10% formalin, processed, and embedded in paraffin by the Northwestern University Mouse Histology and Phenotyping Core facility. The formalin-fixed paraffin-embedded (FFPE) samples were then sectioned (5 μm sections) and mounted on glass slides for staining.

The antibodies used in this study were as follows: rabbit anti-PGP9.5 (1:500, catalog # ab108986, RRID: AB_10891773; abcam, Cambridge, UK) and mouse anti-human mast cell tryptase (1:500, with cross-reactivity with mouse, catalog # 369402, RRID: AB_2566541; BioLegend®, San Diego, CA). The immunolabeling was visualized by using Cy^tm^2-conjugated donkey anti-mouse IgG and Cy^tm^5-conjugated-donkey anti-rabbit IgG and mounted using diamond Antifade mounting medium (Invitrogen, Thermo Fisher, Hampton, NH).

Images were obtained on a Nikon A1 Confocal Laser Microscope System (Plan Apo 20 × NA 0.75 and Plan Apo 60 × NA1.4 Oil, objectives). Images were taken on the same confocal imaging settings as the template for acquiring all images.

### Toluidine Blue Staining

Toluidine blue staining, a metachromatic dye, stain mast cells red-purple (metachromatic staining) and the background blue (orthochromatic staining) for the identification of mast cells. Briefly, 5 μm-thick sections were stained using fresh 0.1% toluidine blue for 2–3 mins, washed thrice using distilled water, dehydrated in 50, 70, 95, and 100% of ethanol, cleared in xylene, and mounted with Krystalon (EMD Millipore, MA, USA). Bright-field images were taken on a Leica DMLA microscope (Leica Microsystems, Buffalo Grove, IL) using a QImaging MicroPublisher 3.3 RTV camera (Teledyne Photometrics, Tucson, AZ) and analyzed on Micromanager, an open-source microscopy software (35).

### Tactile Allodynia Measurement

Animals were tested for pain by quantifying referred visceral pain as cutaneous hyperalgesia. Mice were individually tested for cutaneous hyperalgesia with von Frey filaments (with forces of 0.04, 0.16, 0.4, 1, and 4 g) as previously described (13, 36, 37). Briefly, mice were acclimated in the procedure room for 1 h by placing them in individual plexiglass chambers (6 × 10 × 12 cm) on top of a stainless steel wire grid floor and suspended 2 feet above a flat surface. Calibrated von Frey filaments with forces of 0.04, 0.16, 0.4, 1, and 4 g were used to determine the development of pelvic allodynia. The filaments were applied in increasing force order with a brief resting period between filaments (~5 secs) and filaments were applied 10 times. The filaments were applied to the pelvic area considered located adjacent to the prostate. The criteria for positive response to the filament stimulation were as follows: (1) licking and scratching (hind paws) of the pelvic area immediately after stimulation, (2) retraction of the abdomen, and (3) immediate avoidance (jumping) (37). The testing was performed in a blinded manner. Data are reported as the percentage of positive response ± SEM for each fiber and for all fibers in total.

### Trigeminal Ganglion Extraction and Neuronal Culture

Trigeminal ganglions (TGs) were harvested from C57BL/6 mice, and dissociated sensory neuron cultures were prepared as previously described (38). Briefly, the TGs were excised from the mice, and then placed in ice-cold HBSS without Ca^++^/Mg^++^. TGs were cut into the smaller pieces at the size of ~0.5 mm in a 35 mm petri dish in cold HBSS. After centrifuging at 100 g for 1 min, the tissues were digested in papain, followed by a collagenase/dispase solution at 37°C for 20 mins each. The pellet was collected after centrifuging at 400 g for 4 mins and single cell suspension was prepared in 0.5ml pre-warmed L15 medium (containing 5% FCS, penicillin/streptomycin, HEPES; Fisher Scientific, Hampton, NH). The trigeminal ganglion neurons were obtained after the removal of myelin and tissue debris by using a Percoll gradient centrifuge at 1,300 g for 10 mins. The trigeminal ganglion neurons were then washed with L15 media and then plated and grown in a 24-well plate at 5 × 10^5^ cells per well in pre-warmed complete F12 media (containing 10% FBS, penicillin/streptomycin, HEPES; Fisher Scientific) containing 50 ng/mL NGF and allowed to culture for 6 days. After 6 days of culture, the dissociated sensory neurons were stimulated with mature recombinant mMCP6 prepared above and recombinant mMCP7. TG cultured neurons were lysed for RNA extraction and real-RT2 Profiler^TM^ PCR assay was performed as described below.

### RT2 Profiler™ PCR Array

Total RNA was extracted using RNeasy Mini Kit (Qiagen, Valencia, CA, USA), and cDNA was synthesized using the RT^2^ First Strand Kit (Qiagen) following the manufacturer's instructions. Eighty-four genes or biological intermediates involved in mediating neuropathic and inflammatory pain was analyzed using the RT^2^ Profiler PCR Array Mouse Pain: Neuropathic and Inflammatory. According to the manufacturer's protocol, real-time PCR was performed using RT^2^ Profiler PCR Arrays in combination with RT^2^ SYBR Green/ROX PCR Master Mix (Qiagen). The expression levels were quantified relative to the values obtained for housekeeping genes (Actb, B2m, Gapdh, Gusb, and Hsp90ab1). Data analysis was performed, and clustergram and 2-D scatter plots were generated using Qiagen web-based data analysis software (https://geneglobe.qiagen.com/us/analyze).

### Human Tryptase Delta 1 ELISA

The cytokine ELISA for human δ-tryptase 1 (TPSD1) was performed using EPS samples from 7 controls and 18 CPPS patients with human TPSD1 / Tryptase Delta 1 (Sandwich ELISA) ELISA Kit (LSBio, Seattle, WA, USA). The protein content of EPS was determined by Pierce BCA protein assay (Thermo Fisher, Hampton, NH, USA) and normalized to 1 μg of protein for use in the human δ-tryptase ELISA. The ELISA was performed according to manufacturer's instructions.

### Statistical Analyses

Statistical analyses were performed using GraphPad Prism™ (GraphPad Software, San Diego, CA, USA). Statistical tests utilized in each experiment, technical replicates, biological replicates, independent repeat experiments performed, and murine *n* values are indicated in figure legends. ELISA analysis was performed using a combination of linear regression and correlation (Pearson) between TPDS1 and patient-reported pain scores. Total differences were determined between patients and controls using unpaired *T-*test, with those showing significance subjected to ROC (Receiver Operator Characteristic) analyses. Data are represented as the mean ± standard deviation (SD) or mean ± standard error of the mean (S.E.M.) as appropriate. #*p* < 0.1, ^*^p < 0.05, ^**^p < 0.01, ^***^p < 0.001, ^****^p < 0.0001.

## Results

### Elevated Levels of Human δ Tryptase Is Present in the EPS of CP/CPPS Patients

**Figure 1:**
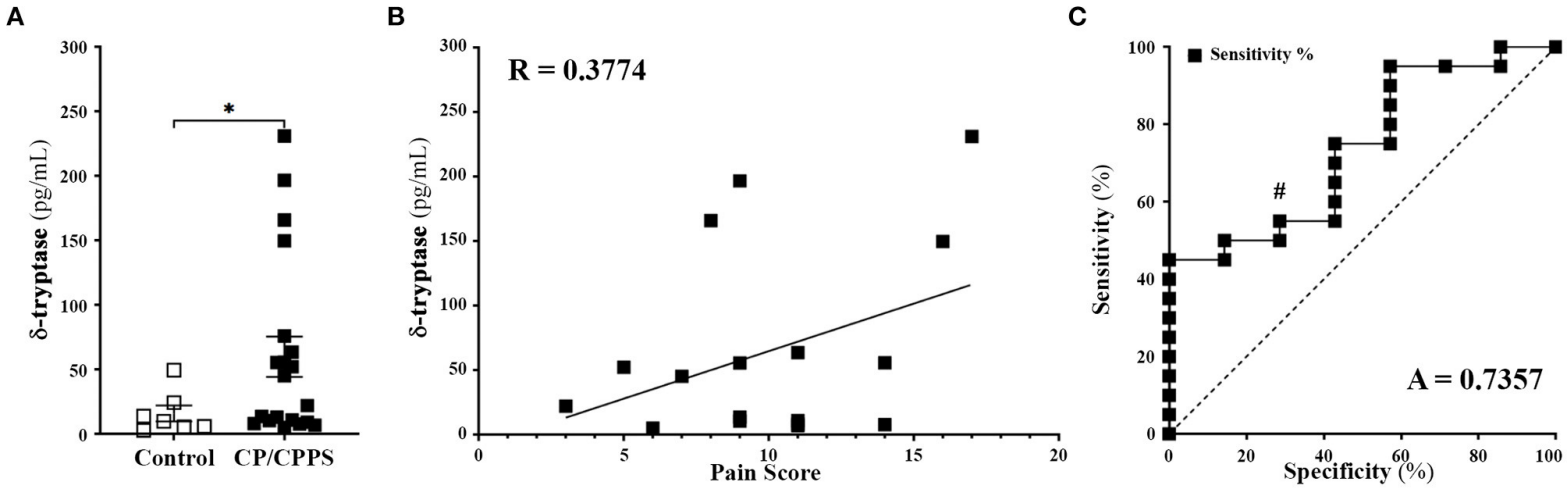
Patients with CP/CPPS have elevated levels of δ tryptase. **(A)** Human δ tryptase (TPSD1) levels measured in EPS from control healthy volunteers (*n* = *7*) and CP/CPPS patients (*n* = 20). Data represented as means ± SEM, each data point represents individual patients; ^*^p < 0.05; unpaired Welch's *t*-test. **(B)** Correlation between increasing patient pain score and levels of δ tryptase in the EPS of patients with CP/CPPS using simple liner regression and R value represents Pearson correlation coefficient. **(C)** ROC curve demonstrating diagnostic power for the use of δ tryptase. A = area under curve, #*p* < 0.1.

We have previously shown that tryptases released from mast cells play a critical role in driving pelvic pain in a mouse model of CPPS, and have also shown that patients with CPPS showed elevated levels of total mast cell tryptase in their EPS (13, 14). In this study, we wanted to examine the levels of human δ-tryptase, an isoform of mast cell tryptase known to be expressed in multiple tissues in humans (22). We compared the levels of δ-tryptase using normalized levels of protein and observed significantly elevated levels in the EPS of CP/CPPS patients compared to healthy controls (Figure 1A). The expression of δ-tryptase was then correlated with the pain sub-score from the National Institutes of Health-Chronic prostatitis symptom index (NIH-CPSI) questionnaires. The linear regression and correlation (Pearson) plots demonstrated that as patient pain sub-scores increased, the presence of δ-tryptase measured in EPS also increased, suggesting a positive correlation between the two parameters (Figure 1B). A ROC analysis was also performed to test the efficacy of this significantly increased δ-tryptase as a diagnostic tool. The ROC analysis revealed an area under the curve value of 0.7357, with sensitivity at 100% and specificity at 14.29% for values above 4.118 pg/mL of δ-tryptase at a significance of p < 0.0679 (Figure 1C). These data suggest a role for δ-tryptase in pain mechanisms in CP/CPPS.

### CP1 *E. Coli* Instillation in C57BL/6J and NOD/ShiLtJ Mice Elevate Mast Cell Numbers in the Prostate but Produce Differential Effects on Pelvic Tactile Allodynia

Our laboratory has previously demonstrated that an intra-urethral instillation of CP1 induces chronic prostate inflammation, fibrosis and urinary dysfunction in both C57BL/6 and NOD/ShiLtJ mice (29–31), but produce pelvic tactile allodynia only in NOD/ShiLtJ not in C57BL/6 mice (29, 31). Here, in addition to confirming that CP1 instillation induces differential pain response between C57BL/6 and NOD/ShiLtJ mice, we examined if there were changes in the presence of mast cells in the prostates between the two strains.

**Figure 2:**
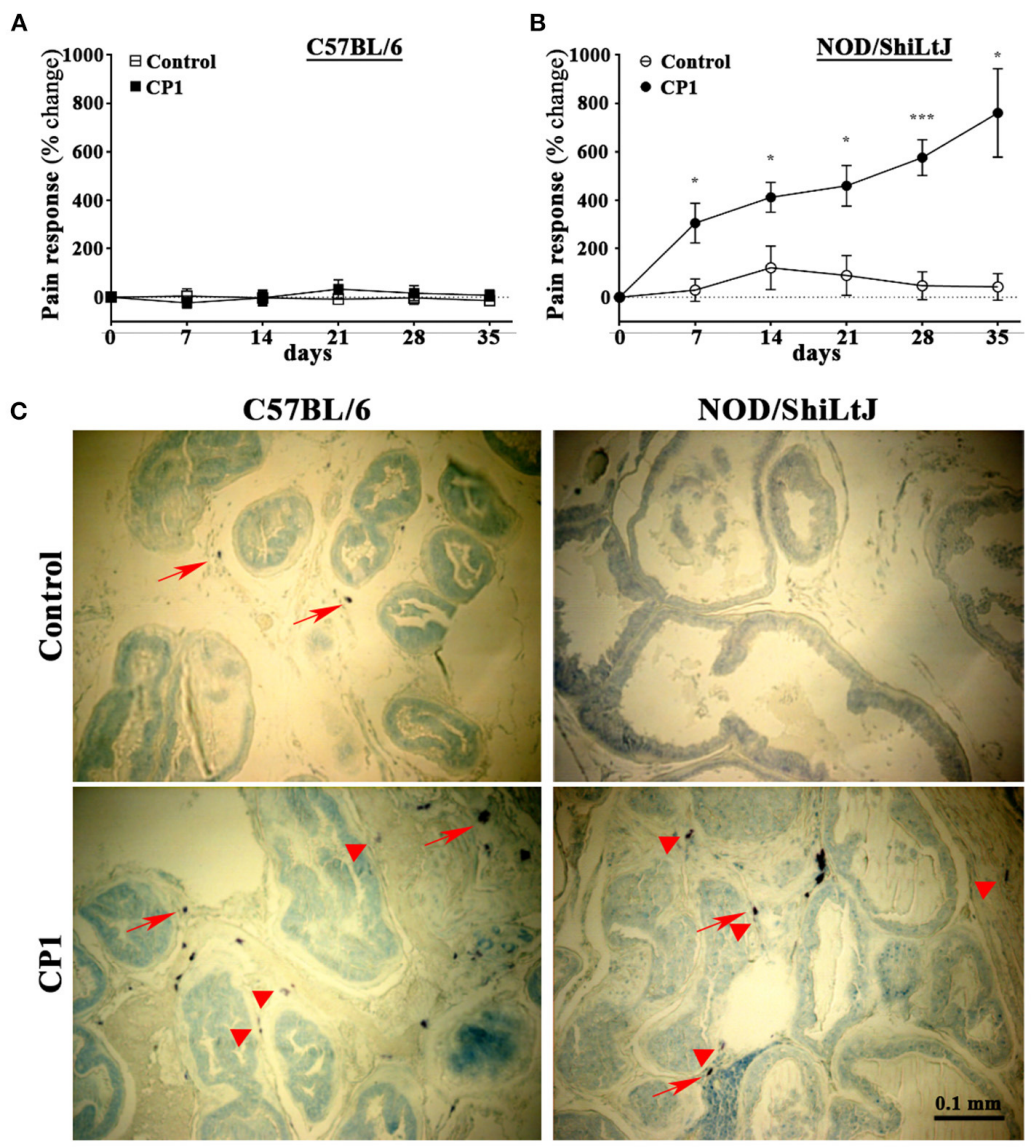
CP1 administration induces mast cells infiltration into the stroma of prostate and differential pelvic tactile allodynia in C57BL/6 and NOD/ShiLtJ mice. **(A, B)** Pelvic tactile allodynia/hyperalgesia was assessed with von Frey filaments of five calibrated forces before (Day 0) and every 7 days till the endpoint of experiment at day 35 post intra-urethral instillation of CP1. Percentage change in pain response was measured in PBS control and CP1 instilled **(A)** C57BL/6 mice and **(B)** NOD/ShiLtJ mice compared to baseline Day 0 (values are presented as means ± SE; *n* = 5 mice/group. *p < 0.05; ***p < 0.001; two-way ANOVA with Fisher's least significant difference test). **(C)** Mast cell-specific toluidine staining showing the presence of mast cells in the stroma of prostates of C57BL/6 and NOD/ShiLtJ mice. Representative images (from *n* = 4–6 mice/group) of toluidine blue-stained dorsolateral prostate sections from control PBS-instilled (top left) and CP1-infected (bottom left) C57BL/6 mice, and control (top right) and CP1-infected (bottom right) NOD/ShiLtJ mice at 35 days post CP1 instillation imaged at 20X magnification. Note the increased degranulation of mast cells (as denoted by less intracytoplasmic granular staining of mast cells) marked by red arrowheads, whereas resting mast cells (with intact cytoplasm and membrane) are marked by red arrows.

CP1 instillation of NOD/ShiLtJ mice showed a significant increase in pelvic tactile allodynia response upon stimulation with Von Frey filaments at days 7, 14, 21, 28, and 35 post instillation compared to controls (Figure 2B). C57BL/6 mice instilled with CP1 at the same time as NOD/ShiLtJ mice showed, no increase in pelvic pain response compared to control mice (Figure 2A).

To assess the presence of mast cells, we examined prostate sections to determine the numbers of mast cells in both C57BL/6 and NOD/ShiLtJ mice upon CP1 instillation. We and others have previously demonstrated that following bacterial instillation, inflammation and mast cell numbers are most pronounced in the dorsolateral lobe of the prostate (29, 39, 40). Dorsolateral prostate lobe sections from CP1-infected mice and control C57BL/6 and NOD/ShiLtJ mice were subjected to toluidine blue staining to assess increase of mast cell numbers. We observed an increase in numbers of mast cells in the dorso-lateral lobes of both C57BL/6 and NOD/ShiLtJ mice upon CP1 instillation compared with control mice (Figure 2C).

### Increased Mast Cell Interaction With Neuronal Structures in the Prostates of CP1-Infected NOD/ShiLtJ Mice

The crosstalk between the immune cells and nervous system is an important feature in the development of inflammatory and neuropathic pain. There have been numerous reports, including in CP/CPPS, describing the bidirectional communication between mast cells and sensory nerve fibers and its potential role in nociception (41–45). Here, we assessed the interactions between mast cells and sensory nerve fibers in the prostates of C57BL/6 and NOD/ShiLtJ mice upon CP1 instillation.

**Figure 3:**
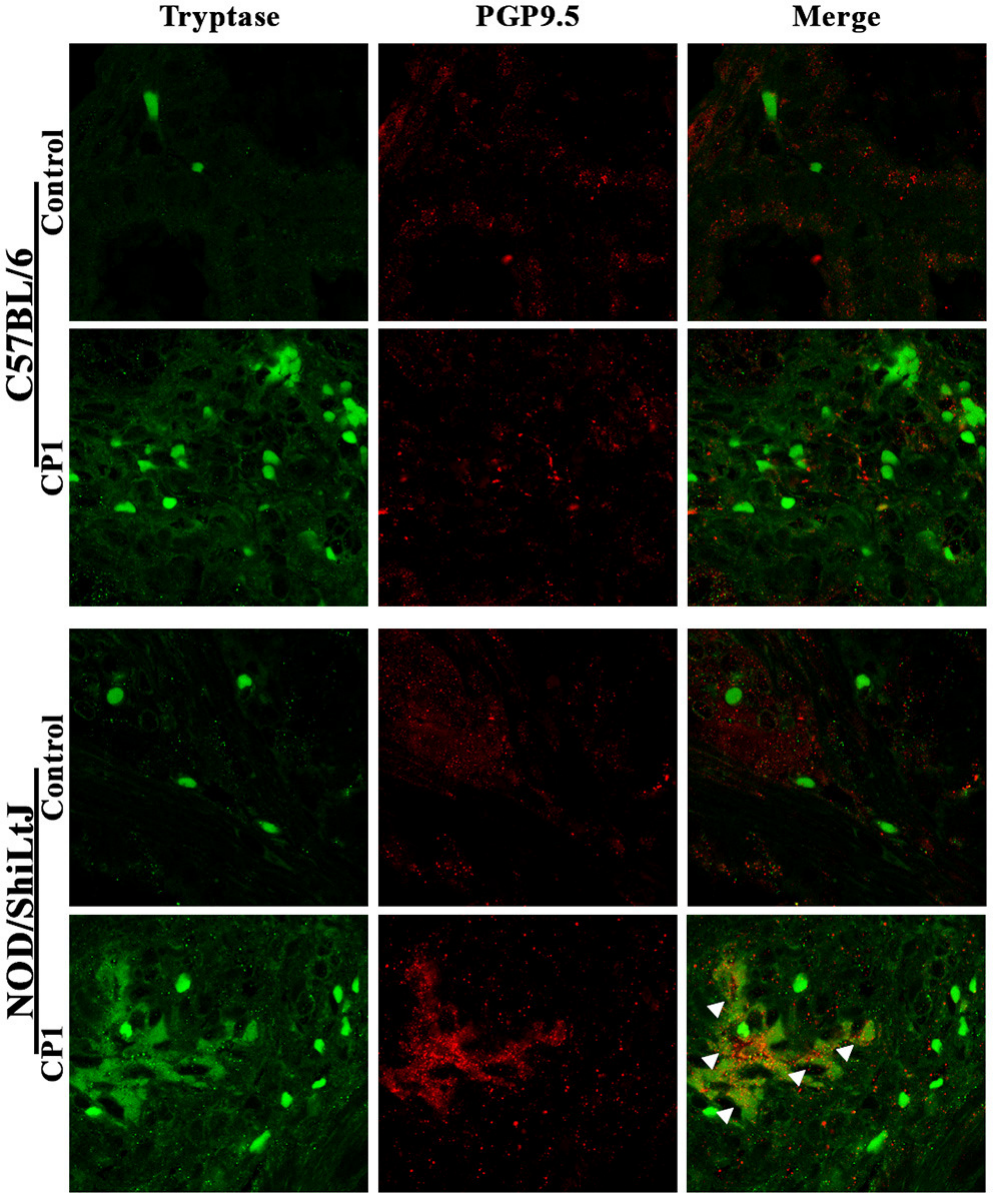
Increased mast cell-nerve interactions in the prostate in NOD/ShiLtJ mice. Representative confocal images (from *n* = 4-6 mice/group) of the dorso-lateral lobe of the prostates from C57BL/6 (upper panels) and NOD/ShiLtJ (bottom panels) mice comparing CP1-infected mice to control mice at day-35 post CP1 instillation. Mast cells are stained using tryptase antibody (green, left panels) in the stroma of the prostates, and nerve fibers that innervate the prostate are stained using PGP9.5 antibody (red, middle panels). A merge of the two channels is used to detect the interactions between tryptase^+ve^ mast cells and PGP9.5 labeled nerve fibers (right panels). White arrowheads denote the cluster of activated mast cells in association with PGP9.5^+ve^ never fibers in the prostate section to form heterogeneous cellular nodules within the region of stroma in NOD/ShiLtJ mice instilled with CP1 (bottom right panel), but not in C57BL/6 mice.

Dorsolateral prostate lobe sections from control or CP1-infected C57BL/6 and NOD/ShiLtJ mice were stained with a pan-tryptase antibody, to stain for mast cells, and anti-PGP9.5 antibody, to identify nerve fibers followed by confocal microscopy. Consistent with Figure 2C, we observed increased mast cell numbers in the prostates of both C57BL/6 and NOD/ShiLtJ mice upon CP1 instillation compared to control mice (Figure 3, left panels). Interestingly, we observed an increase in the staining of PGP9.5^+^ nerve fibers in the prostates of CP1-infected NOD/ShiLtJ mice compared to control mice while we observed no such increase in the prostates of CP1-infected C57BL/6 mice (Figure 3, middle panels). A merge of the two channels shows that a cluster of highly activated and degranulated mast cells aggregated and wrapped around PGP9.5^+^ nerve fibers in the prostate stroma of CP1-infected NOD/ShiLtJ mice (Figure 3, bottom right panel), but not in the prostates of C57BL/6 mice. Our results suggest that increased interactions between activated mast cells and neuronal fibers in the prostates of CP1-infected NOD/ShiLtJ mice, but not C57BL/6, may contribute significantly to the increased sensitization and pelvic tactile allodynia.

### Mouse Ortholog of Human δ-Tryptase, mMCP7 Induces Pelvic Tactile Allodynia in C57BL/6J Mice

While NOD/ShiLtJ and C57BL/6 mice express most of the same mast cell proteases, C57BL/6 mice have a deficiency in the mouse ortholog of δ-tryptase mast cell protease 7 (mMCP7) gene with a point mutation in the exon/intron 2 splice of the mMCP7 gene rendering them unable to express mMCP7 protein (25). Here, in addition to verifying the absence of mMCP7 in C57BL/6 mice, we determined the effect of administration of mMCP7 on pelvic pain in C57BL/6 mice.

**Figure 4:**
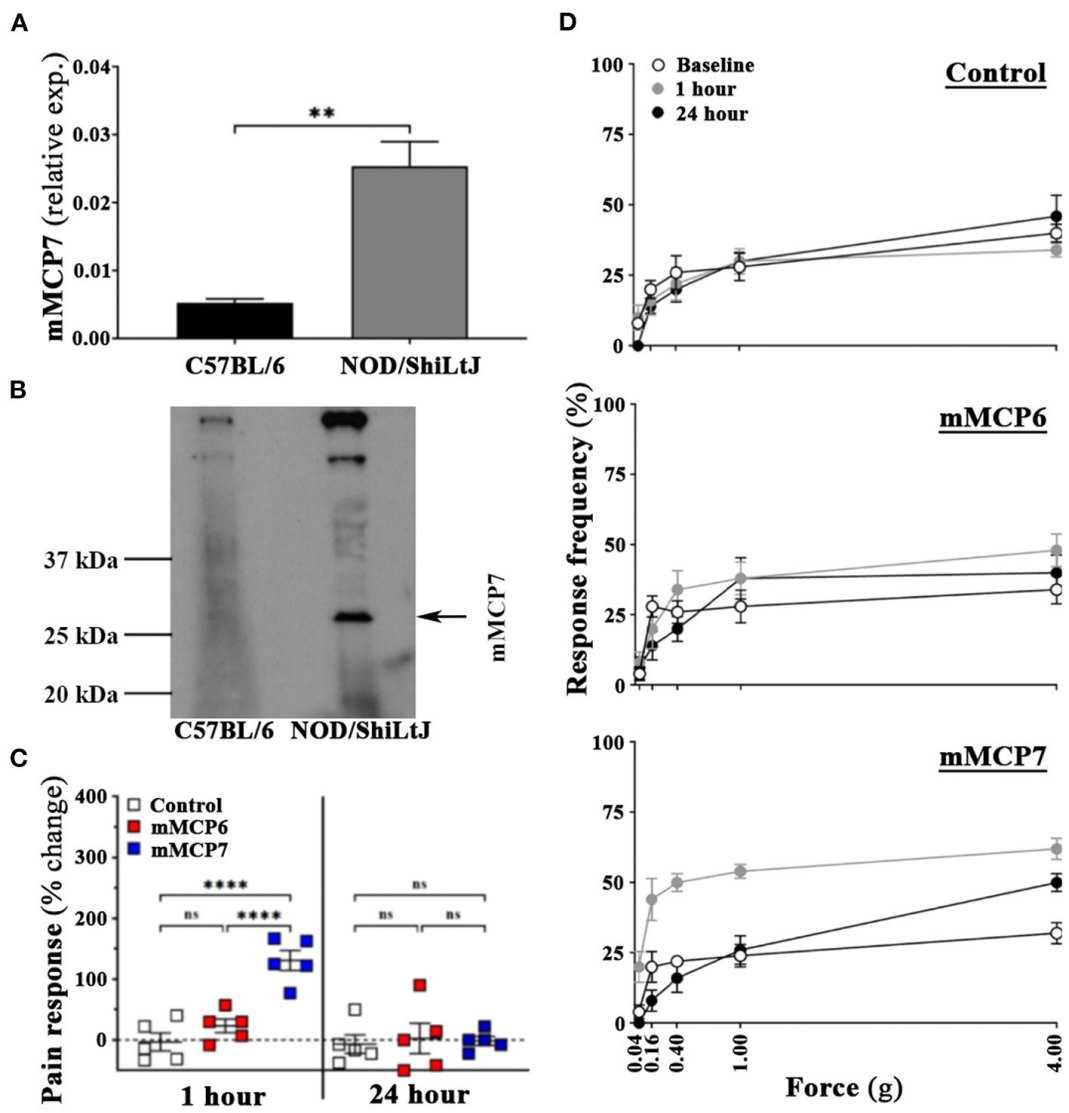
Administration of mMCP7, and not mMCP6, induces pelvic pain in C57BL/6 mice. **(A)** mRNA expression levels for mMCP7 were examined from RNA isolated from ear punch biopsy samples of C57BL/6 and NOD/ShiLtJ mice. RNA was isolated, converted to cDNA, and subjected to real-time PCR analysis with the respective gene primers. mRNA expression levels were normalized to L19 mRNA levels for each sample, and the data are presented as relative expression levels (values are presented as means ± SD; **p < 0.01; unpaired student's *T-*test). **(B)** Total protein levels of mMCP7 examined from prostates of C57BL/6 and NOD/ShiLtJ mice. Whole cell lysates were prepared from total prostates, and 100μg of total lysate were run on a gel and probed for mMCP7 using anti-tryptase alpha/beta 1 antibody. **(C,D)** C57BL/6 mice were assessed for pelvic tactile allodynia/hyperalgesia with von Frey filaments before (baseline) and 1 h and 24 h post intra-urethral instillation of PBS, or mMCP6 (1 ng), or mMCP7 (1ng). **(C)** The change in pain response (%) was generated from the total response frequency at 1 h and 24 h by normalizing it to its respective baseline (values are presented as means ± SE; n = 5 mice/group; each dot represents an individual mouse; ****p < 0.0001; one-way ANOVA Fisher's LSD test; ns, not significant). **(D)** The individual von Frey filament response frequency for each of the experiment groups are shown for baseline, 1 h and 24 h for instillation. Data shown is a representative from three independent experiments.

To determine the differential expression of mMCP7 in C57BL/6 mice, we detected the presence of mMCP7 gene expression levels in the ear biopsy of C57BL/6 and NOD/ShiLtJ mice. NOD/ShiLtJ mice show high levels of gene expression for mMCP7, while C57BL/6 mice show negligible levels of mMCP7 gene expression (Figure 4A). Furthermore, we checked protein expression of mMCP7 in the prostates of C57BL/6 and NOD/ShiLtJ mice using a mMCP7 specific antibody. We observe that prostate lysates from NOD/ShiLtJ mice show high levels of expression of mMCP7, while prostate lysates from C57BL/6 mice show undetectable levels of mMCP7 protein levels (Figure 4B).

Our data so far shows that upon CP1 instillation, prostates from both NOD/ShiLtJ and C57BL/6 mice show increased mast cell numbers, but only NOD/ShiLtJ mice develop pain (Figures 1, 2). Furthermore, C57BL/6 mice do not express mMCP7 (Figures 4A,B). To determine whether the presence of mMCP7 alone can drive enhanced pelvic pain, as measured by tactile allodynia, recombinant mMCP7 and mMCP6 were instilled intra-urethrally in C57BL/6 mice and pelvic tactile allodynia was measured at 1-h and 24-h. C57BL/6 mice that were instilled with recombinant mMCP6 show no significant changes in pain response compared to control mice at both 1-h and 24-h (Figures 4C,D—middle panel). However, C57BL/6 mice that were intra-urethrally instilled with mMCP7 showed a significantly increased change in pain response (>125% pain increase compared to baseline) at 1-h compared to control mice (Figure 4C), and this pain response returned back to baseline levels at 24-h post instillation (Figures 4C,D—bottom panel). This result suggests that mMCP7 administration alone can drive transient pelvic tactile allodynia in C57BL/6 mice.

### mMCP7 Is Capable of Activation of Mast Cells Resulting in Release of Pain Mediators

**Figure 5:**
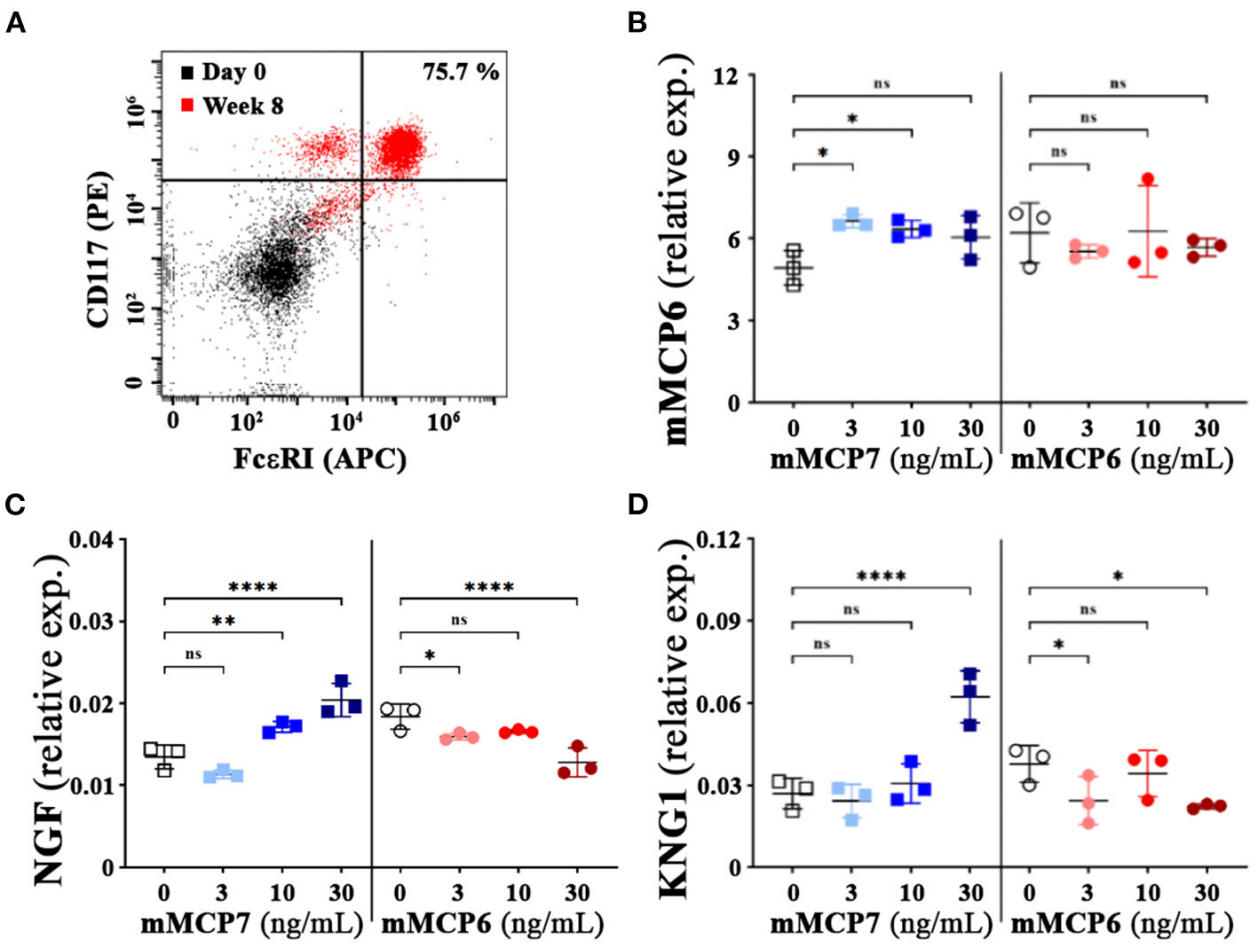
Effect of mMCP7 on mast cell mediated release of neurotrophins and neurotransmitters. **(A)** flow cytometric assessment of bone marrow cells from C57BL/6 mice differentiated to mast cells at 0 week and 8 weeks after differentiation with IL-3 using CD117 (PE) and FcεR1α (APC) (as indicated in MATERIALS AND METHODS). **(B,D)** mRNA expression levels for mMCP6 **(B)**, neurotrophin; NGF **(C)**, and kininogen 1 (KNG1); a precursor for bradykinin **(D)** were examined from RNA isolated from BMMCs treated with different doses of recombinant mMCP6 or mMCP7 for 3 h as indicated. RNA was isolated, converted to cDNA, and subjected to real-time PCR analysis with the respective gene primers. mRNA expression levels were normalized to GAPDH mRNA levels for each sample, and the data are presented as fold changes over control (untreated or control buffer for mMCP7 and mMPC6 respectively). Values are presented as means ± SD; *p < 0.05; **p < 0.01; **** *p* < 0.0001 (one-way ANOVA with Fisher's least significant difference test). ns, not significant.

Activation of mast cells results in the release of pain mediators, including histamine, proteases (e.g., tryptases), serotonins, neurotrophins, neurotransmitters (like bradykinin) and prostaglandin E2, that have been implicated to play a crucial role the development of nociception (4–7). To assess whether mMCP7 or mMCP6 were capable of differential autocrine activation of mast cells, recombinant mMCP7 and mMCP6 were used to treat mast cells derived from bone marrow from C57BL/6 mice (BMMCs). The differentiation of BMMCs as previously described was verified by flow cytometry (32). After 8-weeks of differentiation *in-vitro*, the cells were predominantly double positive for both CD117 and FcεRIα, a distinct identity marker for mast cells (Figure 5A).

To assess the impact of recombinant mMCP6 and mMCP7 on the activation of these differentiated mast cells, quantitative PCR was performed upon treatment with various concentration of the proteases for 3-h. Upon treatment with mMCP7 but not mMCP6, we observed a modest but significant upregulation of mMCP6 gene expression compared to untreated cells (Figure 5B). Furthermore, treatment of these mast cells with mMCP7, but not mMCP6, also caused an upregulation of gene expression of NGF, and kininogen-1 (KNG1); a precursor for the neurotransmitter bradykinin (Figures 5C,D). These data suggest that mMCP7, but not mMCP6, is able to differentially activate mast cells to release pain factors and mediators responsible for interacting with neurons and contributing to the development of nociception.

### mMCP7 Triggers a Unique Expression Signature of Pain-Related Genes in Trigeminal Neurons *in vitro*

**Figure 6:**
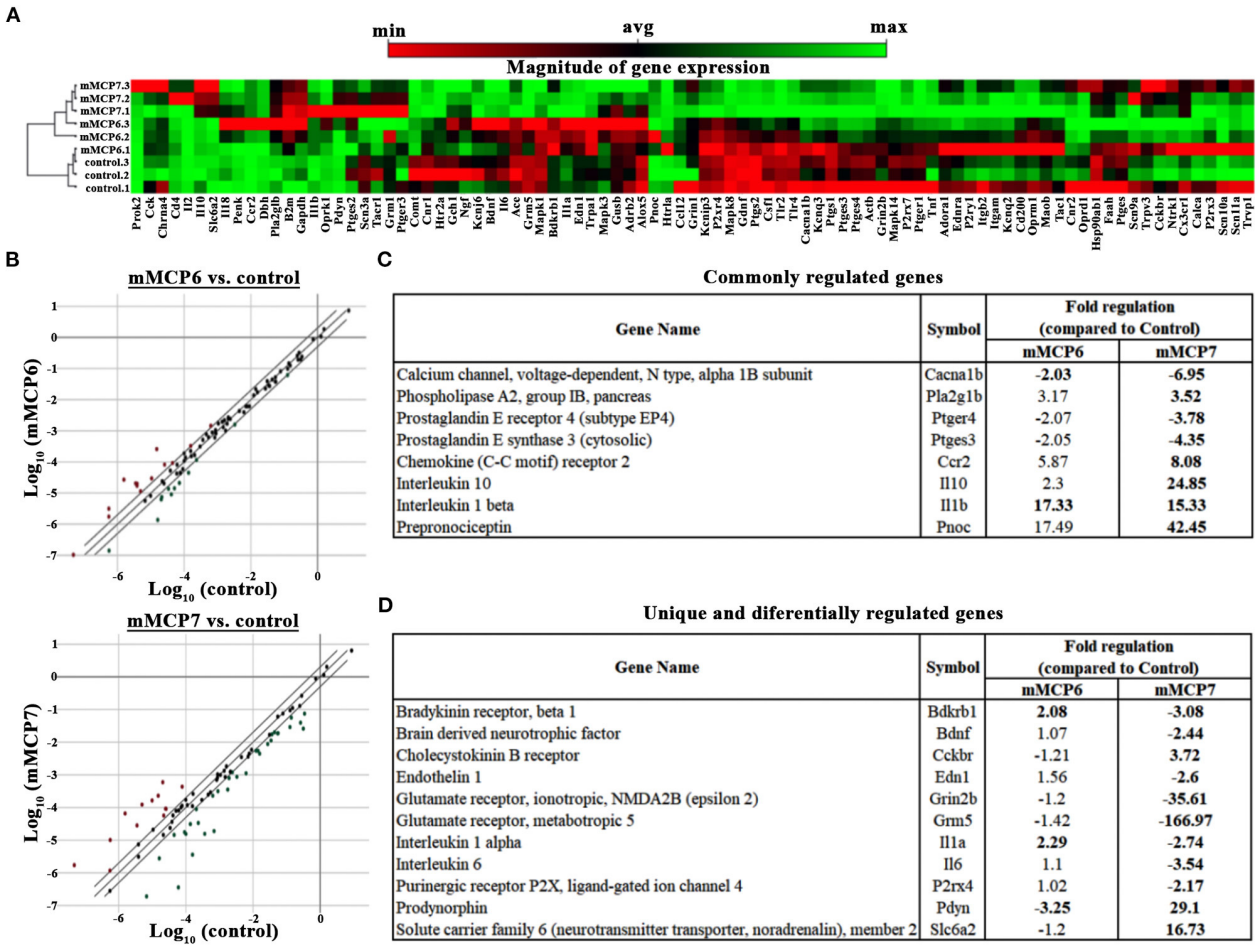
Effect of mMCP7 on transcription of pain-related genes in dissociated trigeminal sensory neurons. Primary mouse dissociated TG neurons were treated with 100 ng/mL of recombinant mMCP6 or mMCP7 for 3 h. RNA was isolated from the neurons, converted to cDNA, and subjected to qRT-PCR using the Qiagen RT2 profiler array for mRNAs of 84 genes related to neuropathic and inflammatory pain. **(A)** Clustergram generated using the Qiagen web-based analysis tool for 3 sets of experiments using mRNA from TG neurons treated with mMCP6 and mMCP7. **(B)** 2-dimensional scatter plot showing the regulation of various genes regulated upon stimulation of TGs with mMCP6 (top panel), and mMCP7 (bottom panel) compared to control. Red dots represent genes that are upregulated and green dots represent genes that are downregulated compared to control. **(C)** A list of genes that are commonly regulated and their fold regulation in both mMCP6 and mMCP7 treated TGs compared to control. **(D)** A list of genes that are unique to, and are differentially expressed in TGs treated with mMCP7 as opposed to TGs treated with mMCP6 and their fold regulation compared to controls. Genes demonstrating a two-fold difference in regulation and a *p* value <0.05 was considered significant. For the qRT-PCR, fold change in gene expression was determined by the 2(–ΔΔCT) method. Statistical significance of the RT2 PCR data was determined using a Student's *t-*test (https://dataanalysis.qiagen.com/pcr/arrayanalysis).

Activation of PAR2 on neurons by mast cell-released tryptases causes neuronal excitability (46). To determine the role played by mMCP6 and mMCP7 on neuronal excitability, primary mouse trigeminal ganglion (TG) neurons from C57BL/6 mice were incubated with recombinant mMCP6 and mMCP7. After treatment with these recombinant proteases, RNA were extracted and RNA array for neuropathic and inflammatory pain was used to assess gene expression changes by qPCR using Qiagen RT2 Profiler Array. Not surprisingly and consistent with the previous results that mMCP6 and mMCP7 regulate pain differentially, the expression pattern for pain-related genes showed heterogeneity between the two proteases (Figures 6A,B).

Upon treatment of TG neuronal culture with mMCP6, neuropathic and inflammatory pain-related genes were significantly regulated (p-value < 0.05) including, bradykinin receptor (Brdkrb1), IL1α, IL1β, and sodium channel 3 (Scn3a) (Table 1). When TG neurons were treated with mMCP7, expression levels of a large number of genes were significantly regulated (p < 0.05) (Table 2). Upon treatment with mMCP7, expression of anti-nociceptive genes such as IL10 and prodynorphin (Pdyn), an opioid peptide precursor, are upregulated in TG neuronal culture, while expression of pro-nociceptive genes like Bdnf, Gdnf, IL1α, IL6, Tlr2, and Tlr4 are downregulated in these TG neuronal cultures. Comparing the effects of these two tryptases mMCP6 and mMCP7, we observe that while there are some commonly regulated genes (Figure 6C), mMCP6 and mMCP7 each have a unique expression signature of pain-related genes (Figure 6D).

**Table 1:** Expression levels of genes significantly regulated in TG neurons upon treatment with mMCP6.

**Gene name**	**Gene symbol**	**Fold regulation (to control)**	***p*** **value**
Bradykinin receptor, beta 1	Bdkrb1	2.08	0.000829
Interleukin 1 alpha	IL1α	2.29	0.021179
Interleukin 1 beta	IL1β	17.33	0.003419
Sodium channel, voltage-gated, type III, alpha	Scn3α	−2.86	0.009923

*Data shown are genes that are significantly regulated (two-fold regulation threshold with p-value < 0.05, Student's T-test, n = 3) in TG neuronal cultures upon treatment with mMCP6 compared to untreated control TG neurons. Data is calculated using the Qiagen web-based analysis portal using ΔΔCT method, using an average of the housekeeping genes for normalization and fold change is calculated compared to controls*.

**Table 2:** Expression levels of genes significantly regulated in TG neurons upon treatment with mMCP7.

**Gene name**	**Gene symbol**	**Fold regulation (to control)**	***p*** **value**
Chemokine (C–C motif) receptor 2	Ccr2	8.08	0.000001
Interleukin 10	IL10	24.85	0.005313
Prodynorphin	Pdyn	29.1	0.025794
Phospholipase A2, group IB, pancreas	Pla2g1b	3.52	0.001123
Solute carrier family 6 (neurotransmitter transporter, noradrenalin), member 2	Slc6a2	16.73	0.002643
Angiotensin I converting enzyme (peptidyl-dipeptidase A) 1	Ace	−2.84	0.003876
Brain derived neurotrophic factor	Bdnf	−2.44	0.006337
Calcium channel, voltage-dependent, N type, alpha 1B subunit	Cacna1b	−6.95	0.045369
Cannabinoid receptor 1 (brain)	Cnr1	−4.98	0.00234
Colony stimulating factor 1 (macrophage)	Csf1	−4.58	0.000819
Endothelin 1	Edn1	−2.6	0.000044
Glial cell line derived neurotrophic factor	Gdnf	−6.59	0.000002
Glutamate receptor, ionotropic, NMDA2B (epsilon 2)	Grin2b	−35.61	0.039252
Glutamate receptor, metabotropic 5	Grm5	−166.97	0.000000
5-hydroxytryptamine (serotonin) receptor 1A	Htr2a	−5.57	0.013452
Interleukin 1 alpha	IL1a	−2.74	0.006165
Interleukin 6	Il6	−3.54	0.01101
Kv channel interacting protein 3, calsenilin	Kcnip3	−2.01	0.002291
Potassium voltage-gated channel, subfamily Q, member 3	Kcnq3	−5.18	0.02401
Mitogen-activated protein kinase 14	Mapk14	−2.88	0.00257
Mitogen-activated protein kinase 8	Mapk8	−3.06	0.000000
Purinergic receptor P2X, ligand-gated ion channel 4	P2rx4	−2.17	0.000432
Purinergic receptor P2X, ligand-gated ion channel, 7	P2rx7	−2.97	0.009561
Prostaglandin E receptor 1 (subtype EP1)	Ptger1	−2.11	0.003776
Prostaglandin E receptor 4 (subtype EP4)	Ptger4	−3.78	0.004387
Prostaglandin E synthase 3 (cytosolic)	Ptges3	−4.35	0.020386
Prostaglandin-endoperoxide synthase 2	Ptgs2	−12.15	0.000000
Toll-like receptor 2	Tlr2	−2.42	0.000015
Toll-like receptor 4	Tlr4	−3.12	0.000233

*Data shown are genes that are significantly regulated (two-fold regulation threshold with p-value < 0.05, Student's T-test, n = 3) in TG neuronal cultures upon treatment with mMCP7 compared to untreated control TG neurons. Data is calculated using the Qiagen web-based analysis portal using ΔΔCT method, using an average of the housekeeping genes for normalization and fold change is calculated compared to controls*.

## Discussion

Chronic Prostatitis/Chronic Pelvic Pain Syndrome (CP/CPPS) accounts for more than 90% of urology clinic visits with a prostatitis diagnoses (1–3, 47). CP/CPPS is a debilitating condition that is marked by both chronic pain and increased inflammatory outcomes with neuropathic/inflammatory pain playing an important role in the progression of the disease (41, 47, 48). In this study, using an *E. coli* (CP1)-induced mouse model of CP/CPPS, we assess the potential role of mMCP7, a mouse ortholog of δ-tryptase in nociception and the development of pelvic tactile allodynia.

Previously our lab has demonstrated an increase in β-tryptase in the EPS of patients with CP/CPPS and examined the role played by β-tryptase in CP/CCPS symptoms (13, 14, 49). In an animal model of CP/CPPS called experimental autoimmune prostatitis (EAP), we have shown the importance of the activation of mast cells and its released tryptases acting on its receptor PAR2 in mediating pain in NOD/ShiLtJ mice. Research in the field of tryptase mediated pain has mostly focused on α/β-tryptases (6), and little attention has been given to the role played by the δ-tryptase isoform, a truncated protein and a supposedly catalytically inactive form (23, 24). Intriguingly, in this study, we observed an elevated level of δ-tryptase in the EPS of patients with CP/CPPS. While we also showed a positive correlation between the severity of the pain sub-score and the levels of δ-tryptase, the biological mechanism and the possible role played by this isoform of tryptase in chronic pain is yet to be elucidated. Interestingly, we observe heterogeneity in the distribution of δ-tryptase in patients with CP/CPPS, with several having levels comparable to that of controls. Previous studies have detected heterogenous levels of β-tryptase, NGF, carboxypeptidase A, IL7, and GROα in the EPS from CP/CPPS patients (13, 14, 18). We did not detect differences in patient parameters between high and low δ-tryptase groups. However, we do not rule out the possibility that the detected heterogeneity may reflect underlying differences in mast cell pathogenesis in patients that may be better identified in a longitudinal study.

Using an uropathogenic *E. coli* (CP1) strain isolated from the prostatic fluid of a patient suffering from CP/CPPS, we have previously established an CP1-induced mouse model of prostatitis in both NOD/ShiLtJ and C57BL/6 mice. Interestingly, only NOD/ShiLtJ mice developed pelvic tactile allodynia (29–31). This suggests, that in this particular model, there is strain-specificity in the development of pelvic tactile allodynia in the NOD/ShiLtJ genetic background, but not in C57BL/6. An important and noteworthy aspect is that in the EAP model of CP/CPPS, both NOD/ShiLtJ and C57BL/6 mice develop pelvic tactile allodynia, albeit the C57BL/6 mice develop pelvic tactile allodynia to a lesser extent (13, 14, 37). In both the EAP and the CP1-induced mouse models of prostatitis, we have shown increased numbers and activity of mast cells in the prostate stroma (13, 14, 29, 39). Furthermore, in the EAP model, we have observed an increase in PGP9.5^+ve^ neuronal structures in the stroma of the prostates (37, 50). In this study, we identified mast cells closely associated with PGP9.5^+ve^ nerve fibers in the prostate of CP1-instilled NOD/ShiLtJ and not in C57BL/6 mice, suggesting for the first time in this model a role for mast cell-mediated neural sensitization in the prostate. Such mast cell-mediated neuronal sensitization has been previously observed in other diseased conditions such as pruritic skin diseases, itch models, migraine, sickle cell pain and vulvodynia (43, 45, 51–54). This difference in the interaction between the mast cells and PGP9.5^+ve^ neuronal structures observed in NOD/ShiLtJ and C57BL/6 mice suggests that cross-talk between these two cell types is critical and may be a precursor to mechanisms driving the pelvic allodynia in NOD/ShiLtJ genetic background, but not in C57BL/6. While the human prostate is anatomically different from the mouse, gene expression pattern analysis has shown that the dorsolateral lobe is closest to that of the human prostate peripheral zone (55). In addition, the transition zone in humans similar to the dorsolateral lobes in mice surrounds the urethra between the bladder and the verumontanum (56). The peripheral and transitional zones of the human prostate may therefore be sites of mast cell mediated effects on chronic pain in CP/CPPS.

While NOD/ShiLtJ and C57BL/6 mice have a number of genetic differences between the two strains of mice (57), one gene of interest that is absent in the C57BL/6 strain but is present in the NOD/ShiLtJ strain is the mouse ortholog of δ-tryptase, mMCP7 gene (26, 27). The C57BL/6 mice has a point mutation in the exon/intron 2 splice of the mMCP7 gene resulting in a loss of expression of mMCP7 protein (25). Both these strains of mice express mMCP6 gene. While human α- and β-tryptases are orthologs of mMCP6, the ancestral MCP7 ortholog was replaced by parts of other tryptases, creating δ-tryptase which has high similarity to mMCP7 (24). Thus, the CP1-induced prostatitis model induced difference in pelvic tactile allodynia provides us with a unique and novel means of studying the role played by δ-tryptase in CP/CPPS. Interestingly, a single intra-urethral administration of mMCP7 causes pelvic tactile allodynia in C57BL/6 mice suggesting the possibility that the presence of mMCP7 in the NOD/ShiLtJ mice is a major factor in the development of pelvic tactile allodynia in the CP1-induced model of prostatitis.

Mast cells are a multi-functional cell type that play key roles in the pathophysiological processes like host defense, regulation of vascular tone and permeability, angiogenesis, cell recruitment, tissue remodeling, and neural activities (58). Mast cells modulate these biological activates by degranulation and the release of factors including histamines, TNFα, carboxypeptidase A, tryptases, serotonins, dopamine, chymases, VEGF, and NGF (8, 10, 59–64). mMCP7 is able to act directly on mast cells and trigger its activation independent of degranulation (data not shown). In an autocrine fashion, it is possible that the increase in mMCP7 in the NOD/ShiLtJ mice upon CP1 intra-urethral instillation triggers a release of neurotrophins and neuropeptides from mast cells to facilitate its interactions with the neuronal structures. This mMCP7-mediated interaction, which is absent in C57BL/6, is a possible mechanism for the development of pelvic tactile allodynia.

Our understanding of the cross-talk between mast cells and neurons in the prostate is limited. However, we do know that mast cell tryptases act through PAR2 on neurons to induce neuronal excitability (46). Indeed, neurons treated with mMCP6 show an increase in markers of neuronal excitability. Surprisingly, while neurons treated with mMCP7 show activation and differential effects on pain-related molecules, the markers predominantly increased are the anti-inflammatory cytokine IL-10, and prodynorphin, an opioid molecule; and markers of neuronal excitability like Bdnf, Gdnf, and pro-inflammatory cytokines like IL-1α, IL-6 are down-regulated. This pattern of gene regulation by mMCP7 is suggestive of a pain-related compensatory mechanism like those described previously in acute pain and spinal injury (65, 66). This observation provides another layer of complexity in the possible role played by mMCP7 (and maybe δ-tryptase) in regulating chronic pain.

Histopathological studies have shown limited correlation between the presence of leukocytes and clinical symptoms in CP/CPPS (67). The CP1 infection model in the C57BL/6 vs NOD/ShiLtJ mice provides a mechanistic explanation for this enigma by demonstrating that the mere presence of inflammation is not a determining factor for the development of pain. Rather, the unique signature of released factors (like δ-tryptase from mast cells) might be the differentiator that determines whether susceptible men develop clinical symptoms in CP/CPPS patients.

In summary, mast cells appear to play a critical role in the development of pelvic tactile allodynia through the action of mMCP7, a mast cell protease that has similarities to the human ortholog δ-tryptase. mMCP7 is capable of inducing a unique signature of inflammation and nociception associated molecules through autocrine and paracrine functions on mast cells and neurons that may contribute to nociception. Relevance of these findings to clinical CP/CPPS lies both in examining the role of this unexplored δ-tryptase isoform is chronic pain pathogenesis as well as in its utility as a potential biomarker to identify and differentiate CP/CPPS from underlying inflammatory conditions.

## Data Availability Statement

The original contributions presented in the study are included in the article/supplementary material, further inquiries can be directed to the corresponding author/s.

## Ethics Statement

The studies involving human participants were reviewed and approved by Northwestern University Institutional Review Board Panel D. The patients/participants provided their written informed consent to participate in this study. The animal study was reviewed and approved by Institutional Animal Care and Use Committee (IACUC) at Northwestern University.

## Author Contributions

GP, ZL, and PT: conceived and designed research, analyzed data, and interpreted results of experiments. GP, ZL, and MP: performed experiments. GP and ZL: prepared figures and drafted the manuscript. GP, ZL, AS, and PT: edited, and finalized the manuscript. All authors approved the final version of the manuscript.

## Funding

This work was supported by the National Institute of Diabetes and Digestive and Kidney (NIDDK) Grant Nos. R01DK083609 and R01DK124460. The funders had no role in study design, data collection and analysis, decision to publish, or preparation of manuscript.

## Conflict of Interest

The authors declare that the research was conducted in the absence of any commercial or financial relationships that could be construed as a potential conflict of interest.

## Publisher's Note

All claims expressed in this article are solely those of the authors and do not necessarily represent those of their affiliated organizations, or those of the publisher, the editors and the reviewers. Any product that may be evaluated in this article, or claim that may be made by its manufacturer, is not guaranteed or endorsed by the publisher.
